# Association of oral status with frailty among older adults in nursing homes: a cross-sectional study

**DOI:** 10.1186/s12903-023-03009-8

**Published:** 2023-06-07

**Authors:** Siyue Liu, Yicong Guo, Zhao Hu, Feixiang Zhou, Shaojie Li, Huilan Xu

**Affiliations:** Department of Social Medicine and Health Management, Xiangya School of Public Health, Central South University, Changsha, 410078 China

**Keywords:** Frailty, Oral health, The frequency of brushing tooth, Nursing homes, Older adults

## Abstract

**Background:**

The oral status of an individual is a vital aspect of their overall health. However, older adults in nursing homes have a higher prevalence of frailty and poor oral health, particularly in the context of global aging. The objective of this study is to explore the association between oral status and frailty among older adults residing in nursing homes.

**Methods:**

The study involved 1280 individuals aged 60 and above from nursing homes in Hunan province, China. A simple frailty questionnaire (FRAIL scale) was used to evaluate physical frailty, while the Oral Health Assessment Tool was used to assess oral status. The frequency of tooth brushing was classified as never, once a day, and twice or more a day. The traditional multinomial logistic regression model was used to analyze the association between oral status and frailty. Adjusted odds ratios (*OR*) and 95% confidence intervals (*CI*) were estimated while controlling for other confounding factors.

**Results:**

The study found that the prevalence of frailty among older adults living in nursing homes was 53.6%, while the prevalence of pre-frailty was 36.3%. After controlling for all potential confounding factors, mouth changes requiring monitoring (*OR* = 2.10, 95% *CI* = 1.34–3.31, *P* = 0.001) and unhealthy mouth (*OR *= 2.55, 95% *CI *= 1.61–4.06, *P* < 0.001) were significantly associated with increased odds of frailty among older adults in nursing homes. Similarly, both mouth changes requiring monitoring (*OR *= 1.91, 95% *CI* = 1.20–3.06, *P* = 0.007) and unhealthy mouth (*OR* = 2.24, 95% *CI* = 1.39–3.63, *P* = 0.001) were significantly associated with a higher prevalence of pre-frailty. Moreover, brushing teeth twice or more times a day was found to be significantly associated with a lower prevalence of both pre-frailty (*OR *= 0.55, 95% *CI* = 0.34–0.88, *P* = 0.013) and frailty (*OR* = 0.50, 95% *CI *= 0.32–0.78, *P* = 0.002). Conversely, never brushing teeth was significantly associated with higher odds of pre-frailty (*OR* = 1.82, 95% *CI* = 1.09–3.05, *P* = 0.022) and frailty (*OR* = 1.74, 95% *CI* = 1.06–2.88, *P* = 0.030).

**Conclusions:**

Mouth changes that require monitoring and unhealthy mouth increase the likelihood of frailty among older adults in nursing homes. On the other hand, those who brush their teeth frequently have a lower prevalence of frailty. However, further research is needed to determine whether improving the oral status of older adults can change their level of frailty.

**Supplementary Information:**

The online version contains supplementary material available at 10.1186/s12903-023-03009-8.

## Background

As society advances and medical technology improves, the global population of older adults is on the rise. In fact, according to the seventh national census, aging is a basic national condition in China. The proportion of Chinese individuals aged 65 and above has reached 13.50%, which surpasses the global average [1]. However, the traditional function of Chinese home care for the older adults is no longer adequate to meet the growing demand due to changes in family structure. Specifically, families are increasingly adopting the “4-2-1” family structure, where four older adults are supported by a couple and one child, leading to a decline in traditional home care for the older adults [2].

The increasing number of nursing homes aims to provide relief for home care, as more older adults may choose to enter these facilities in the future. However, it is worth noting that a majority of older people who are admitted to nursing homes have serious physical illnesses that require nursing staff assistance with basic daily activities [3]. In fact, over 10% of older adults in nursing homes have two or more comorbidities [4]. The percentage of older adults in nursing homes with limitations in activities of daily living and instrumental activities of daily living is high, at 82.9% and 89.4%, respectively [5]. Additionally, about 32.4% of older adults in nursing homes experience severe cognitive impairment [6]. Thus, the health status of older adults in nursing homes should not be overlooked, and they require more attention to promote their physical and mental well-being, as well as their ability to adapt to society.

Frailty has recently emerged as a prominent focus in the field of modern geriatric medicine. Frailty is a clinical medical syndrome that is characterized by an increased susceptibility to stressors, and it results from reduced or dysfunctional physiological reserves in multiple physiological systems [7]. The prevalence of frailty is higher among older adults, especially those living in nursing homes, where it can reach up to 52.3%[8]. In China, the prevalence of frailty among nursing home residents is reported to be 44.3%[9]. Numerous studies have highlighted the significant association between frailty in older adults and a range of adverse outcomes, including falls [10], disability [11], mortality [12], depression [13], and others.

Oral status is an essential component of an individual’s overall health. However, older adults living in nursing homes generally have poorer oral health compared to their counterparts [14]. For instance, a study conducted in Shanghai nursing homes revealed that older adults reported poor oral health related to tongue health, saliva production, natural tooth retention, and oral cleanliness [15]. Moreover, poor oral status has been linked to several adverse health outcomes in older adults. Studies have established a strong association between poor oral status and depressive symptoms [16], health-related quality of life [17], burden on healthcare services [18], and mortality [19].

It is crucial to explore the prevalence and associated factors of poor oral health in frail older adults to mitigate their suffering and negative consequences. Previous research has identified various factors linked to poor oral status in older adults, including but not limited to nutritional status [20], the number of medications taken [21], depression [22], loneliness, and disability [23]. While many studies have explored the association between oral status and frailty in community-dwelling older adults, limited research has investigated the association between oral status and frailty among older adults in nursing homes, particularly in China.

Focusing on the association between oral status and frailty among older adults in nursing homes is both important and necessary. Not only can improving oral status help reduce the risk of adverse outcomes at the individual level, but providing oral health education and interventions can also benefit frail older adults on the nursing care level. Such interventions can enhance their quality of life and promote their physical and mental health. Therefore, the aim of this study is to investigate the association between oral status and frailty among older adults living in nursing homes. The findings of this study will provide a basis for relevant departments and personnel to develop oral care programs for frail older adults.

## Methods

### Participants

A cross-sectional study was conducted in nursing homes across Hunan Province, China from July 2021 to April 2022. Informed consent was obtained from all participants. A multi-stage sampling method was employed to select a representative sample of older adults residing in nursing homes in Hunan Province. First, we selected one city each from western, northern, southern, and central Hunan based on region: Xiangxi Autonomous Prefecture city, Yongzhuo city, Yiyang city, and Changsha city. We then randomly selected half of the counties/districts from each selected city. Next, we included all nursing homes located in each selected county/district, which resulted in a total of 22 nursing homes. Finally, we recruited all eligible older adults living in the selected nursing homes for this study.

This study included older adults living in selected nursing homes who met the following inclusion criteria: (1) were 60 years or older; (2) had been living in the nursing home for at least three months; and (3) were able to communicate normally and voluntarily agreed to participate in the study. However, older adults who met any of the following exclusion criteria were excluded from the study: (1) were unconscious or in a coma; (2) had a severe illness such as stage IV heart failure, tumors with multiple metastases, or other serious organic diseases; (3) had audio-visual impairments or language communication difficulties; or (4) had Alzheimer’s disease or other forms of dementia.

A total of 2104 older adults were recruited for this study. After excluding 310 participants who lived in the nursing homes less than 3 months, and 514 were diagnosed with Alzheimer’s disease, a total of 1280 participants were finally included.

### Measurements

#### Physical frailty assessment

The simple frailty questionnaire (FRAIL scale) was used to assess physical frailty of the participants in this study [24]. This scale includes questions about fatigue, resistance, ambulation, illnesses, and weight loss. The Chinese version of FRAIL scale was validated by Dong et al. [25]. And the results showed that it had good reliability and validity to assess the frailty among older adults in China.

The FRAIL scale comprises 5 specific questions, including: “How much time did you feel tired during the past 4 weeks?”, “Do you have any difficulty walking up 10 steps alone without resting and without aids?”, “Do you have any difficulty walking several hundred yards alone and without aids?”, “Do you report 5 or more illnesses out of 11 total illnesses?”, and “Did they report with a weight decline of 5% or greater within the past 12 months?”. Each question is scored on a scale of 0–1 point. Resistance and ambulation items are scored in reverse, while the others are scored positively. The total scores are accumulated by adding the scores for all 5 items, with a range of 0 to 5 points. Physical pre-frailty is indicated when the total score on the FRAIL scale is 1–2 points, while physical frailty is indicated when the total score is 3 points or above.

#### Oral health assessment

In this study, qualified students majoring in stomatology were responsible for collecting data on the oral hygiene status of older adults in nursing homes, after following systematic training under the guidance of dentists. The Oral Health Assessment Tool (OHAT) was used to evaluate the oral health of the participants. The OHAT was revised by Chalmers et al. and has been shown to be suitable for assessing oral health in all kinds of older adults [26]. This validated tool assesses various aspects of oral health, including the lips, tongue, gums and tissues, saliva, natural teeth, dentures, oral cleanliness, and toothache. The Chinese version of OHAT results indicated that the OHAT has good reliability and validity, with a Cronbach’s α coefficient of 0.71 and a reliability of 0.811 [27]. And it can be used as an oral health assessment tool for older adults in China.

The OHAT assessment involves assigning a score of 0, 1, or 2 to each item, depending on the observed condition. A score of 0 represents a healthy state, indicating the absence of any disorder. A score of 1 signifies changes, indicating the presence of a noticeable but non-pathological change, while a score of 2 denotes an unhealthy state, indicating the presence of pathological features.

OHAT total score ranges from 0 to 16 point. This score is classified into three categories for ease of interpretation [15, 28]: (1) 0–3 points indicates healthy mouth: can be maintained through usual care; (2) 4–8 points signifies mouth changes requiring monitoring: observed changes and highlights areas of weakness that require monitoring; (3) 9–16 points denotes unhealthy mouth: care needs to be planned and the specialized opinion of a dental surgeon should be proposed.

#### Brushing frequency Assessment

To gather information about the participants’ brushing habits, we asked the question, “How many times a day do you brush your teeth?” Based on their responses, participants were categorized into one of the following groups: (1) never brushed teeth, (2) brushed teeth once a day, (3) brushed teeth twice or more times a day.

#### Characteristics of nursing homes

In our study, we gathered data on various characteristics of nursing homes, including their geographical location (urban/township), operating model (public-operated/public-private/private-owned), type of institution (with built-in medical facilities/without medical facilities/medical care facility for the older adults), size of institution (< 100 people and ≥ 100 people), frequency of cultural and recreational activities organized (< 2 times/week and ≥ 2 times/week), and the availability of fitness equipment and spaces (yes/ no).

#### Covariates

Several factors have been identified as being related to frailty among older adults, such as smoking [29], drinking [30], napping [31], and pain [32]. To control for potential confounding variables, socio-demographic information, lifestyle behaviors, and health-related conditions were included as covariates. Socio-demographic information comprised age, sex, residence, education, marital status, source of income, income, and number of children. Education levels were classified as illiteracy, primary school, middle school and above. Marital status was divided into married, widowed, and others. Previous studies have shown that most older adults in Chinese nursing homes have a family income of 2000–5000 Ren Min Bi/month (RMB/month)[33], so income was divided into three groups: ≤2000 RMB/month, 2001–5000 RMB/month, and ≥ 5001 RMB/month. Lifestyle behavior covariates included smoking history (never/former/current), drinking history (never/current), and napping (Yes/No) in the past month. Health-related conditions included the number of medicines taken (0/1 ~ 4/≥5), pain (yes/no), and nutrition. Nutrition was assessed using the Mini-Nutritional Assessment Short-Form (MNA-SF scale) [34], which has been validated in the Chinese population with excellent test characteristics [35]. The participants were categorized into three groups according to their total scores: 12–14 points represented well-nourished individuals, 8–11 points represented those at risk of malnutrition, and 0–7 points represented malnourished individuals.

#### Statistical methods

To assess multicollinearity, the variance inflation factor (VIF) was used, where VIF > 10 indicates the presence of multicollinearity. To examine whether there is group aggregation of frailty status in nursing homes, a generalized linear mixed model was employed. The model used the individual as level 1 and the nursing homes where the participant resided as level 2. An intraclass correlation coefficient (ICC) was obtained by establishing a null model to determine whether frailty required analysis using a multilevel model. If the ICC is less than 5%, then second-level aggregation can be disregarded, and a traditional multinomial logistic regression model can be used to investigate the association between oral status and frailty status and to evaluate the goodness of fit of the model.

The analysis of the data followed the following procedures. Categorical variables were presented as frequencies and percentages, while continuous variables were expressed as mean ± standard deviation (SD). To compare the distribution of continuous variables, one-way analysis of variance (ANOVA) was used. The relationship between categorical variables was assessed using the chi-square test. The models were divided into three categories: Model^a^ (unadjusted for confounding factors), Model^b^ (adjusted for socio-demographic information factors), and Model^c^ (adjusted for socio-demographic information factors, lifestyle behaviors and health-related conditions factors). Adjusted odds ratios (*OR*) and 95% confidence intervals (*CI*) were estimated for each model, and statistical significance was determined using *p* < 0.05. All statistical analyses were conducted using Stata version 17.0.

## Results

### Characteristics of nursing homes

Table 1 presents the characteristics of the nursing homes included in this study. The majority of nursing homes were located in urban areas, and 63.6% of them were publicly constructed and operated. Additionally, 36.4% of the nursing homes had built-in medical institutions, while 50.0% had medical care facilities for older adults. Most nursing homes had the capacity to accommodate more than 100 people and offered fitness equipment and spaces. Moreover, 68.2% of the nursing homes organized cultural and recreational activities at least twice a week.

**Table 1:** Characteristics of nursing homes according to the frailty status groups (n = 22)

Characteristics	Number of pension institutions (n)	Composition ratio (%)
Geographic location (n, %)		
Urban	15	68.2
Township	7	31.8
Operating model (n, %)		
Public-operated	14	63.6
Public-private	6	27.3
Private-owned	2	9.1
Type of institution (n, %)		
Built-in medical institutions	8	36.4
No medical institution	3	13.6
Medical care facility for the older adults	11	50.0
Size of institution (people) (n, %)		
< 100	5	22.7
≥ 100	17	77.3
Frequency of cultural and recreational activities organized (n, %)		
<twice a week	7	31.8
≥twice a week	15	68.2
Fitness equipment and spaces (n, %)		
No	2	9.1
Yes	20	90.9

### Characteristics of non-frailty, pre-frailty and frailty in older adults

In this study, all 1280 participants were investigated, with a mean age (± SD) of 77.64 (± 9.87) years, and 53.0% of them were women. The characteristics of the participants based on their frailty status are presented in Table 2. The prevalence of frailty in nursing homes was 53.6%, while 36.3% of participants had pre-frailty. Among older adults with frailty, 47.7% were men and 52.3% were women, while 44.8% of pre-frail older adults were male and 55.2% were female. The majority of pre-frail and frail older adults lived in urban areas before entering the nursing homes. In terms of education level, 43.4% of participants had completed primary school, and 39.9% of pre-frail and 43.9% of frail older adults had primary education. Most older adults were widowed, with the proportion of widows among pre-frail and frail older adults accounting for 62.9% and 48.7%, respectively. The income of pre-frail and frail older adults in nursing homes ranged from 2001 to 5000 Ren Min Bi (RMB), accounting for 87.1% and 69.7%, respectively. Additionally, 61.0% of pre-frail and 65.6% of frail older adults had at least two children.

**Table 2:** Characteristics of the participants according to the frailty status groups (n = 1280)

Characteristics	Overall (n = 1280)	Non-frail (n = 130)	Pre-frail (n = 464)	Frail (n = 686)	*P*-value
Age (years) *	77.64 ± 9.87	74.29 ± 10.27	77.80 ± 9.85	78.18 ± 9.69	0.128**
Age (years) (n, %)					< 0.001
60–69	329 (25.7)	54 (41.5)	118(25.4)	157 (22.9)	
70–79	332 (25.9)	30 (23.1)	130(28.0)	172 (25.1)	
≥ 80	619 (48.4)	46 (35.4)	216(46.6)	357 (52.0)	
Sex (n, %)					0.418
Male	601 (47.0)	66(50.8)	208(44.8)	327 (47.7)	
Female	679 (53.0)	64(49.2)	256(55.2)	359 (52.3)	
Residence (n, %)					0.001
Urban	846 (66.1)	83(63.8)	337(72.6)	426 (62.1)	
Rural	434 (33.9)	47(36.2)	127(27.4)	260 (37.9)	
Education (n, %)					< 0.001
Illiteracy	511 (39.9)	34 (26.2)	220(47.4)	257 (37.5)	
Primary school	555 (43.4)	69 (53.1)	185(39.9)	301 (43.9)	
Middle school and above	214 (16.7)	27 (20.8)	59(12.7)	128 (18.7)	
Marital status (n, %)					< 0.001
Widowed	690 (53.9)	64 (49.2)	292(62.9)	334 (48.7)	
Married	142 (11.1)	20 (15.4)	44(9.5)	78 (11.4)	
Others	448 (35.0)	46 (35.4)	128(27.6)	274 (39.9)	
Source of income (n, %)					0.661
Pensions	919 (71.8)	98 (75.4)	323(69.6)	498 (72.6)	
Family supports	262 (20.5)	22(16.9)	102(22.0)	138 (20.1)	
Others	99 (7.7)	10(7.7)	39(8.4)	50 (7.3)	
Income (n, %)					< 0.001
≤ 2000 RMB	208 (16.3)	17 (13.1)	24(5.2)	167 (24.3)	
2001–5000 RMB	983 (76.8)	101 (77.7)	404(87.1)	478 (69.7)	
≥ 5001 RMB	89 (7.0)	12 (9.2)	36(7.8)	41 (6.0)	
Number of children (n, %)					0.054
0	165 (12.9)	23 (17.7)	57(12.3)	85 (12.4)	
1	296 (23.1)	21(16.2)	124(26.7)	151 (22.0)	
≥ 2	819 (64.0)	86 (66.2)	283(61.0)	450 (65.6)	
Nutrition (n, %)					< 0.001
Well-nourished	598 (46.7)	95(73.1)	151(32.5)	352 (51.3)	
At risk of malnutrition	540 (42.2)	30(23.1)	254(54.7)	256 (37.3)	
Malnourished	142(11.1)	5(3.8)	59(12.7)	78 (11.4)	
Number of medicine taken (n, %)					< 0.001
0	303 (23.7)	37 (28.5)	85(18.3)	181 (26.4)	
1 ~ 4	424 (33.1)	41 (31.5)	141(30.4)	242 (35.3)	
≥ 5	553 (43.2)	52 (40.0)	238(51.3)	263 (38.3)	
Pain (n, %)					< 0.001
No	611 (47.7)	72 (55.4)	187(40.3)	352 (51.3)	
Yes	669 (52.3)	58 (44.6)	277(59.7)	334 (48.7)	
Smoking history (n, %)					< 0.001
Never	716 (55.9)	93 (71.5)	288(62.1)	335 (48.8)	
Formal	391 (30.5)	26 (20.0)	126(27.2)	239 (34.8)	
Current	173 (13.5)	11 (8.5)	50(10.8)	112 (16.3)	
Drinking history (n, %)					< 0.001
Never	1003 (78.4)	120 (92.3)	375(80.8)	508 (74.1)	
Current	277 (21.6)	10 (7.7)	89(19.2)	178 (25.9)	
Napping (n, %)					< 0.001
No	784 (61.3)	74 (56.9)	256(55.2)	454 (66.2)	
Yes	496 (38.8)	56 (43.1)	208(44.8)	232 (33.8)	
Oral health (n, %)					0.001
Healthy mouth	365 (28.5)	58 (44.6)	130(28.0)	177(25.8)	
Mouth changes requiring monitoring	445 (34.8)	38 (29.2)	163(35.1)	244 (35.6)	
Unhealthy mouth	470 (36.7)	34 (26.2)	171(36.9)	265 (38.6)	
Brushing frequency (n, %)					< 0.001
Twice or more times a day	219 (17.1)	39 (30.0)	76(16.4)	104(15.2)	
Once a day	674 (52.7)	68 (52.3)	240(51.7)	366 (53.4)	
Never	387 (30.2)	23 (17.7)	148(31.9)	216 (31.5)	

*Data are presented as the mean ± standard deviation (SD)

**p-values were obtained from analysis of variance (ANOVA).

The participants were classified into three groups based on their status of frailty, and Table 2 provides further information on the variations between them. The results of the chi-squared test indicated significant differences in several aspects such as age, residence, education, marital status, income, nutrition, the number of medications taken, pain, smoking and drinking history, napping, oral health, and brushing frequency.

### Characteristics of the subdomains of OHAT for older adults with non-frailty, pre-frailty and frailty

In this study, the subdomains of OHAT were compared among older adults with different status of frailty, as presented in Table 3. The findings indicated significant differences among the groups in the subdomains of saliva (*P* = 0.011), natural teeth (*P* = 0.026), dentures (*P* = 0.009), and toothache (*P* = 0.006).

**Table 3:** Characteristics of the subdomains of HOAT according to the frailty status groups (n = 1280)

Items	Overall (n = 1280)	Non-frail (n = 130)	Pre-frail (n = 464)	Frail (n = 686)	*P*-value
Lips (n, %)					0.196
Healthy mouth	550(42.9)	57(43.8)	219(47.2)	274(39.9)	
Mouth changes requiring monitoring	469(36.6)	47(36.2)	156(33.6)	266(38.8)	
Unhealthy mouth	261(20.4)	26(20.0)	89(19.2)	146(21.3)	
Tongue (n, %)					0.060
Healthy mouth	555(43.3)	58(44.6)	225(48.5)	272(39.7)	
Mouth changes requiring monitoring	521(40.7)	51(39.2)	174(37.5)	296(43.1)	
Unhealthy mouth	204(15.9)	21(16.2)	65(14.0)	118(17.2)	
Gums and tissues (n, %)					0.068
Healthy mouth	334(26.1)	38(29.2)	137(29.5)	159(23.2)	
Mouth changes requiring monitoring	548(42.8)	50(38.5)	200(43.1)	298(43.4)	
Unhealthy mouth	398(31.1)	42(32.3)	127(27.4)	229(33.4)	
Saliva (n, %)					0.011
Healthy mouth	455(35.5)	55(42.3)	184(39.7)	216(31.5)	
Mouth changes requiring monitoring	605(47.3)	59(45.4)	198(42.7)	348(50.7)	
Unhealthy mouth	220(17.2)	16(12.3)	82(17.7)	122(17.8)	
Natural tooth (n, %)					0.026
Healthy mouth	406(31.7)	46(35.4)	159(34.3)	201(29.3)	
Mouth changes requiring monitoring	556(43.4)	52(40.0)	212(45.7)	292(42.6)	
Unhealthy mouth	318(24.8)	32(24.6)	93(20.0)	193(28.1)	
Dentures (n, %)					0.009
Healthy mouth	494(38.6)	54(41.5)	205(44.2)	235(34.3)	
Mouth changes requiring monitoring	484(37.8)	49(37.7)	152(32.8)	283(41.3)	
Unhealthy mouth	302(23.6)	27(20.8)	107(23.1)	168(24.5)	
Oral cleanliness (n, %)					0.050
Healthy mouth	320(25.0)	35(26.9)	132(28.4)	153(22.3)	
Mouth changes requiring monitoring	687(53.7)	68(52.3)	250(53.9)	369(53.8)	
Unhealthy mouth	273(21.3)	27(20.8)	82(17.7)	164(23.9)	
Ttoothache (n, %)					0.006
Healthy mouth	408(31.9)	39(30.0)	174(37.5)	195(28.4)	
Mouth changes requiring monitoring	616(48.1)	67(51.5)	215(46.3)	334(48.7)	
Unhealthy mouth	256(20.0)	24(18.5)	75(16.2)	157(22.9)	

### Association of oral status with frailty status

After conducting the multicollinearity analysis, the results revealed that all variables’ VIF values were less than 10, indicating the absence of multicollinearity among the variables. Furthermore, the null model of the multinomial logistic regression analysis showed an ICC value of 3.93%, indicating a low level of aggregation effect and the absence of hierarchical structure characteristics in the data. There was no similarity or aggregation observed in the frailty of older adults in different nursing homes, and the hierarchical structure could be disregarded. Therefore, it was appropriate to use the conventional multinomial logistic regression model for analysis.

#### Association of oral health with pre-frailty and frailty

Table 4 presents the results of the multinomial logistic regression model analyzing the association between oral health and frailty status. And the results demonstrated satisfactory goodness of fit. The associations of other covariates with frailty status in oral health Model^a^ and Model^b^ are displayed in Supplementary Tables 1 and Supplementary Tables 2, respectively.

**Table 4:** Associations of oral health with frailty status according to unadjusted and adjusted logistic regression models (n = 1208)

	Non-frail	Pre-frail			Frail		
		*OR*	95% *CI*	*p*-values	*OR*	95% *CI*	*P*-values
**Model** ^a^							
Healthy mouth	Reference	Reference			Reference		
Mouth changes requiring monitoring	Reference	1.79	1.08–2.97	0.024	2.26	1.39–3.66	0.001
Unhealthy mouth	Reference	2.09	1.25–3.50	0.005	2.58	1.57–4.23	< 0.001
**Model** ^**b**^							
Healthy mouth	Reference	Reference			Reference		
Mouth changes requiring monitoring	Reference	1.74	1.07–2.84	0.027	2.22	1.39–3.55	0.001
Unhealthy mouth	Reference	2.16	1.32–3.56	0.002	2.54	1.58–4.10	< 0.001
**Model** ^**c**^							
Healthy mouth	Reference	Reference			Reference		
Mouth changes requiring monitoring	Reference	1.91	1.20–3.06	0.007	2.10	1.34–3.31	0.001
Unhealthy mouth	Reference	2.24	1.39–3.63	0.001	2.55	1.61–4.06	< 0.001

Model ^a^: unadjusted;

Model ^b^: adjusted for age, sex, residence, education, marital status, economic source, income, and number of children;

Model ^c^: adjusted for age, sex, residence, education, marital status, economic source, income, number of children, nutrition, number of medicines taken, pain, smoking history, drinking history, and napping;

In Model^a^, the odds ratio for mouth changes requiring monitoring was 1.79 (95% *CI* = 1.08–2.97, *P* = 0.024) for pre-frail and 2.26 (95% *CI* = 1.39–3.66, *P* = 0.001) for frail, compared with the non-frail group. The likelihood of being pre-frail was 2.09 times for unhealthy mouth than for healthy mouth (*OR* = 2.09, 95% *CI* = 1.25–3.50, *P* = 0.005), and it increased to 2.58 (*OR*=2.58, 95% *CI* = 1.57–4.23, *P* < 0.001) for frailty. After adjusting for all socio-demographic factors in Table 4, mouth changes requiring monitoring (*OR* = 1.74, 95% *CI* = 1.07–2.84, *P* = 0.027) and unhealthy mouth (*OR* = 2.16, 95% *CI* = 1.32–3.56, *P* = 0.002) were significantly associated with an increased odds ratio of pre-frailty. Similar results were found in the frailty group (mouth changes requiring monitoring: *OR* = 2.22, 95% *CI* = 1.39–3.55, *P* = 0.001; unhealthy mouth: *OR* = 2.54, 95% *CI* = 1.58–4.10, *P* < 0.001). Finally, after adjusting for all confounding factors in Table 4, the study indicates a significant association between oral health and pre-frailty or frailty among older adults. The study showed that mouth changes requiring monitoring were associated with a higher prevalence of pre-frailty (*OR* = 1.91, 95% *CI* = 1.20–3.06, *P* = 0.007) and frailty (*OR* = 2.10, 95% *CI* = 1.34–3.31, *P* = 0.001). Unhealthy mouth had a higher odds ratio of pre-frailty (*OR* = 2.24, 95% *CI* = 1.39–3.63, *P* = 0.001) and frailty (*OR* = 2.55, 95% *CI* = 1.61–4.06, *P* < 0.001) compared to healthy mouth in the non-frail group.

#### Association of brushing teeth frequency with pre-frailty and frailty

Table 5 displays the results of the multinomial logistic regression model, which assessed the association between brushing teeth frequently and frailty status. The goodness of fitwas confirmed. Supplementary Tables 3 and Supplementary Table 4 show the associations of other covariates with frailty status in the Model^a^ and Model^b^ of frequent teeth brushing.

**Table 5:** Associations of brushing frequency with frailty status according to unadjusted and adjusted logistic regression models (n = 1208)

	Non-frail	Pre-frail			Frail		
		*OR*	95% *CI*	*P*-values	*OR*	95% *CI*	*P*-values
**Model** ^a^							
Twice or more times a day	Reference	0.59	0.35–0.98	0.040	0.44	0.27–0.72	0.001
Once a day	Reference	Reference			Reference		
Never	Reference	1.95	1.13–3.35	0.017	1.73	1.02–2.93	0.042
**Model** ^**b**^							
Twice or more times a day	Reference	0.56	0.34–0.92	0.022	0.45	0.28–0.71	0.001
Once a day	Reference	Reference			Reference		
Never	Reference	1.86	1.10–3.16	0.021	1.70	1.02–2.84	0.042
**Model** ^**c**^							
Twice or more times a day	Reference	0.55	0.34–0.88	0.013	0.50	0.32–0.78	0.002
Once a day	Reference	Reference			Reference		
Never	Reference	1.82	1.09–3.05	0.022	1.74	1.06–2.88	0.030

Model^a^: unadjusted;

Model^b^: adjusted for age, sex, residence, education, marital status, economic source, income, and number of children;

Model^c^: adjusted for age, sex, residence, education, marital status, economic source, income, number of children, nutrition, number of medicines taken, pain, smoking history, drinking history, and napping;

Initially, brushing teeth twice or more times a day was associated with a decreased odds ratio of both pre-frailty (*OR* = 0.59, 95% *CI* = 0.35–0.98, *P* = 0.040) and frailty (*OR* = 0.44, 95% *CI* = 0.27–0.72, *P* = 0.001) before adjusting for all confounding factors. On the other hand, never brushing teeth was associated with an increased odds ratio of both pre-frailty (*OR* = 1.95, 95% *CI* = 1.13–3.35, *P* = 0.017) and frailty (*OR* = 1.73, 95% *CI* = 1.02–2.93, *P* = 0.042). After adjusting for all socio-demographic factors in Table 5, brushing teeth twice or more times a day was associated with a lower prevalence of both pre-frailty (*OR* = 0.56, 95% *CI* = 0.34–0.92, *P* = 0.022) and frailty (*OR* = 0.45, 95% *CI* = 0.28–0.71, *P* = 0.001), while never brushing teeth was associated with a higher prevalence of both pre-frailty (*OR* = 1.86, 95% *CI* = 1.10–3.16, *P* = 0.021) and frailty (*OR* = 1.70, 95% *CI* = 1.02–2.84, *P* = 0.042). After adjusting for all confounding factors in Table 5, individuals who reported brushing their teeth twice or more times a day had lower odds ratios of both pre-frailty (*OR* = 0.55, 95% *CI* = 0.34–0.88, *P* = 0.013) and frailty (*OR* = 0.50, 95% *CI* = 0.32–0.78, *P* = 0.002) compared to those who brush once a day. Additionally, never brushing teeth was associated with higher odds ratios of both pre-frailty (*OR* = 1.82, 95% *CI* = 1.09–3.05, *P* = 0.022) and frailty (*OR* = 1.74, 95% *CI* = 1.06–2.88, *P* = 0.030).

## Discussion

This study is the first to investigate the link between oral status and frailty among older adults living in nursing homes. Our results showed that both mouth changes requiring monitoring and unhealthy mouth were significantly associated with a higher prevalences of pre-frailty and frailty compared to non-frail older adults in nursing homes. Furthermore, our study suggests that brushing teeth twice or more times a day is associated with a lower prevalence of frailty and pre-frailty, whereas never brushing teeth is linked to a higher prevalence of these conditions.

Our study found that the prevalence of frailty and pre-frailty was 53.6% and 36.3%, respectively, among older adults in nursing homes. These findings suggest a large potential population of frail older adults in nursing homes. A study conducted in Changsha, reported even higher prevalence rates of frailty (60.3%) and pre-frailty (36.2%) among older adults in nursing homes, as assessed by the Fried frailty phenotype scale [36]. In contrast, another study by using FRAIL scale to assess frailty in nursing homes in Shandong found a prevalence of frailty at 29.2% among 370 older adults [37]. Differences in the measurements used to assess frailty and the population of older adults investigated could be possible reasons for the variation in results among Chinese nursing homes. Given that frailty can be reversible [38], early screening and effective interventions are essential for improving or reversing frailty in older adults living in nursing homes.

This study examined the association between impaired oral health and frailty among older adults residing in nursing homes. Although few studies have explored the association between oral health and frailty among older adults in nursing homes, community-dwelling older adults with poor oral hygiene have a high prevalence of frailty [39, 40]. For instance, a study from Taiwan found that frailty was associated with OHAT scores and saliva items in the subdomains of OHAT among community older adults [41]. Another study conducted by Rapp et al. using the same measurement to assess oral health showed a significant association between worsening oral health and frailty among older adults in France [42]. Therefore, appropriate oral health measures should be taken to prevent or reverse frailty in nursing home residents with impaired oral health.

This study observed that older adults in nursing homes who brushed their teeth more frequently had a lower prevalence of pre-frailty and frailty, while those who never brushed their teeth had a higher prevalence. The current studies have focused little on older adults living in nursing homes, a recent study of older adults living in Chinese nursing homes found that regular tooth brushing as an indicator of oral health could reduce the risk of frailty [43]. But some studies have examined the association of brushing frequency with frailty among community-dwelling older adults. For instance, Tuuliainen et al. found that the brushing frequency among Finnish frail older inhabitants remained significantly lower than in the non-frail older adults, and positive changes in the prevalence of brushing teeth twice a day were observed [44]. In 2022, a study from communities in South Korea found that brushing after all three meals was negatively correlated with frailty among older adults aged 50 years or older [45]. Additionally, insufficient brushing has been shown to frailty-related enabling factors [46]. However, a Dutch study showed that brushing teeth was not associated with frailty among older adults, which may be due to different methods of collecting brushing data [47]. While brushing frequency has been linked to frailty to some extent, it is worth noting that frailty may have an influence on the teeth brushing habits of older adults living in nursing homes. These individuals often have limited physical and cognitive abilities, and are more susceptible to muscle weakness, which can result in a reduced frequency of daily tooth brushing. Given the current studies, the field of brushing frequency, whether it is associated with prevalence of frailty among older adults, still needs further studies.

Several studies have shown the pathogenesis between impaired oral health and frailty as the oral status affects multiple domains. Previous studies have suggested that nutrition may play an important role in the association of oral status and frailty among older adults [39, 48]. Common oral problems such as tooth loss, toothache, and dysphagia among older adults can lead to changes in their dietary habits, or even increase the risk of malnutrition [49–51]. A review study reported an association between frailty and intakes of protein, energy, and specific micronutrients [52]. Poor oral cleansing among older adults who do not brush their teeth frequently can result in more plaque or fewer teeth, leading to a higher prevalence of frailty [44]. The increased risk of frailty is often associated with oral diseases caused by failure to clean the mouth in time after consuming sugar-laden drugs and other substances [53]. Despite nursing staff in nursing homes recognizing the importance of oral hygiene for the healthcare of older adults, most have limited oral care skills [54].

The prevalence of frailty among older adults in nursing homes is high, particularly among those with poor oral status. As oral status can be improved, and frailty can be alleviated or reversed, effective intervention is necessary to promote the health of older adults. On one hand, managing oral hygiene in frail older adults requires nursing staff to enhance their skills and educate older adults on oral health care. This approach can improve the oral status of older adults. On the other hand, addressing underlying health problems, maintaining good nutrition, engaging in regular exercise, and seeking out more social support can promote good oral health and reduce the risk of frailty.

However, our study has several limitations. Firstly, this was a cross-sectional study, and a causal relationship between oral status and frailty cannot be inferred. Secondly, self-reported questionnaires were used, and the data obtained may have had some recall bias. Finally, the study only included participants from nursing homes in Hunan province, so the results may not be generalizable to older adults in other nursing homes in China.

## Conclusion

In conclusion, the study determined that mouth changes requiring monitoring, unhealthy mouth, and never brushing teeth were significantly correlated with pre-frailty and frailty among older adults living in nursing homes. Conversely, older adults in nursing homes who brush their teeth more frequently showed a decrease in the prevalence of pre-frailty and frailty. As a result, it is critical for managers and nursing staff to recognize the significance of maintaining good oral health as an essential component of healthcare. Nursing homes should establish a comprehensive training program for nursing staff in oral care skills and use the platform to promote oral health awareness among older adults in nursing homes.

## Electronic supplementary material

Below is the link to the electronic supplementary material.

Supplementary Material 1

## Data Availability

The datasets used and/or analysed during the current study available from the corresponding author on reasonable request.

## Abbreviations

FRAIL: A simple frailty questionnaire
OHAT: Oral Health Assessment Tool
RMB: Ren Min Bi
MNA-SF: Mini-nutritional Assessment Short-Form
