# Supplementary material for: Association of oral status with frailty among older adults in nursing homes: a cross-sectional study
Source: BMC Oral Health. 2023 Jun 7;23:368. doi: 10.1186/s12903-023-03009-8 (PMC10249201; doi:10.1186/s12903-023-03009-8)
Supplement: Supplementary file 1 — Supplementary Material 1 [file 12903_2023_3009_MOESM1_ESM.docx]

**supplementary table：**

**Supplementary Table 1 Associations of other covariates with frailty status in oral health model^a^ (n=1208)**

|  | **Pre-frail** | | | **Frail** | | |
| --- | --- | --- | --- | --- | --- | --- |
|  | **OR** | **95%CI** | ***p*-values** | **OR** | **95%CI** | ***p*-values** |
| Age (years) |  |  |  |  |  |  |
| 70-79 | 1.86 | 1.05-3.27 | 0.033 | 1.94 | 1.12-3.35 | 0.018 |
| ≥80 | 2.04 | 1.16-3.58 | 0.013 | 3.55 | 2.05-6.14 | <0.001 |
| Sex |  |  |  |  |  |  |
| Male | 0.72 | 0.45-1.16 | 0.179 | 0.70 | 0.45-1.10 | 0.118 |
| Residence | . | .. | . |  |  |  |
| Urban | 1.45 | 0.83-2.53 | 0.196 | 1.71 | 0.99-2.93 | 0.053 |
| Education |  |  |  |  |  |  |
| Primary school | 0.53 | 0.32-0.88 | 0.015 | 0.70 | 0.42-1.16 | 0.164 |
| Middle school and above | 0.32 | 0.16-0.62 | 0.001 | 0.61 | 0.32-1.17 | 0.136 |
| Marital status |  |  |  |  |  |  |
| Married | 0.45 | 0.18-1.12 | 0.087 | 0.78 | 0.32-1.88 | 0.581 |
| Others | 1.12 | 0.67-1.86 | 0.668 | 2.05 | 1.26-3.34 | 0.004 |
| Source of income |  |  |  |  |  |  |
| Family supports | 1.53 | 0.80-2.89 | 0.196 | 1.67 | 0.90-3.11 | 0.104 |
| Others | 4.39 | 1.40-13.83 | 0.011 | 1.41 | 0.48-4.18 | 0.534 |
| Income |  |  |  |  |  |  |
| ≥5001 RMB | 0.69 | 0.32-1.49 | 0.346 | 0.54 | 0.25-1.15 | 0.109 |
| ≤2000 RMB | 0.47 | 0.19-1.13 | 0.092 | 3.42 | 1.56-7.49 | 0.002 |
| Number of children |  |  |  |  |  |  |
| 0 | 0.94 | 0.38-2.28 | 0.887 | 0.78 | 0.33-1.82 | 0.562 |
| ≥2 | 0.44 | 0.23-0.84 | 0.013 | 0.65 | 0.35-1.21 | 0.170 |
| Nutrition |  |  |  |  |  |  |
| At risk of malnutrition | 4.12 | 2.48-6.85 | <0.001 | 2.09 | 1.26-3.46 | 0.004 |
| Malnourished | 6.29 | 2.36-16.74 | <0.001 | 4.50 | 1.71-11.90 | 0.002 |
| Number of medicine taken |  |  |  |  |  |  |
| 1~4 | 1.10 | 0.59-2.02 | 0.771 | 0.89 | 0.50-1.57 | 0.682 |
| ≥5 | 1.59 | 0.85-2.97 | 0.147 | 0.77 | 0.42-1.39 | 0.380 |
| Pain |  |  |  |  |  |  |
| Yes | 0.89 | 0.55-1.45 | 0.652 | 0.66 | 0.41-1.05 | 0.082 |
| Smoking history |  |  |  |  |  |  |
| Formal | 1.25 | 0.70-2.24 | 0.458 | 2.09 | 1.19-3.69 | 0.010 |
| Current | 1.56 | 0.71-3.42 | 0.264 | 2.96 | 1.41-6.20 | 0.004 |
| Drinking history |  |  |  |  |  |  |
| Current | 1.68 | 0.78-3.64 | 0.184 | 2.11 | 1.00-4.45 | 0.049 |
| Napping |  |  |  |  |  |  |
| Yes | 1.08 | 0.69-1.68 | 0.736 | 0.56 | 0.36-0.85 | 0.007 |

Reference: taking non-frail group as the reference.

Model^a^: unadjusted;

**Supplementary Table 2 Associations of other covariates with frailty status in oral health model^b^ (n=1208)**

|  | **Pre-frail** | | | **Frail** | | |
| --- | --- | --- | --- | --- | --- | --- |
|  | **OR** | **95%CI** | ***p*-values** | **OR** | **95%CI** | ***p*-values** |
| Nutrition |  |  |  |  |  |  |
| At risk of malnutrition | 4.62 | 2.87-7.45 | <0.001 | 1.90 | 1.19-3.04 | 0.007 |
| Malnourished | 7.18 | 2.74-18.77 | <0.001 | 4.62 | 1.79-11.96 | 0.002 |
| Number of medicine taken |  |  |  |  |  |  |
| 1~4 | 1.19 | 0.68-2.10 | 0.540 | 0.89 | 0.53-1.50 | 0.661 |
| ≥5 | 1.57 | 0.92-2.70 | 0.101 | 0.85 | 0.51-1.42 | 0.540 |
| Pain |  |  |  |  |  |  |
| Yes | 1.03 | 0.66-1.62 | 0.883 | 0.72 | 0.47-1.11 | 0.137 |
| Smoking history |  |  |  |  |  |  |
| Formal | 1.08 | 0.64-1.83 | 0.770 | 2.13 | 1.29-3.53 | 0.003 |
| Current | 1.34 | 0.65-2.80 | 0.429 | 2.43 | 1.22-4.83 | 0.012 |
| Drinking history |  |  |  |  |  |  |
| Current | 1.72 | 0.82-3.62 | 0.151 | 2.61 | 1.28-5.35 | 0.009 |
| Napping |  |  |  |  |  |  |
| Yes | 1.09 | 0.72-1.65 | 0.687 | 0.59 | 0.40-0.88 | 0.010 |

Reference: taking non-frail group as the reference.

Model^b^: adjusted for age, sex, residence, education, marital status, economic source, income, and number of children;

**Supplementary Table 3 Associations of other covariates with frailty status in brushing teeth frequency model^a^ (n=1208)**

|  | **Pre-frail** | | | **Frail** | | |
| --- | --- | --- | --- | --- | --- | --- |
|  | **OR** | **95%CI** | ***p*-values** | **OR** | **95%CI** | ***p*-values** |
| Age (years) |  |  |  |  |  |  |
| 70-79 | 1.83 | 1.03-3.23 | 0.039 | 1.91 | 1.10-3.31 | 0.021 |
| ≥80 | 1.99 | 1.13-3.50 | 0.017 | 3.44 | 1.99-5.98 | <0.001 |
| Sex |  |  |  |  |  |  |
| Male | 0.74 | 0.46-1.18 | 0.201 | 0.71 | 0.45-1.11 | 0.133 |
| Residence | . | .. | . |  |  |  |
| Urban | 1.43 | 0.81-2.50 | 0.214 | 1.67 | 0.97-2.87 | 0.064 |
| Education |  |  |  |  |  |  |
| Primary school | 0.49 | 0.29-0.83 | 0.007 | 0.65 | 0.40-1.08 | 0.098 |
| Middle school and above | 0.30 | 0.15-0.59 | <0.001 | 0.58 | 0.31-1.10 | 0.097 |
| Marital status |  |  |  |  |  |  |
| Married | 0.50 | 0.20-1.24 | <0.001 | 0.91 | 0.38-2.18 | 0.840 |
| Others | 1.15 | 0.69-1.91 | 0.007 | 2.14 | 1.31-3.50 | 0.002 |
| Source of income |  |  |  |  |  |  |
| Family supports | 1.55 | 0.82-2.92 | 0.180 | 1.69 | 0.91-3.13 | 0.097 |
| Others | 4.55 | 1.46-14.18 | 0.009 | 1.51 | 0.52-4.44 | 0.450 |
| Income |  |  |  |  |  |  |
| ≥5001 RMB | 0.78 | 0.35-1.69 | 0.524 | 0.61 | 0.28-1.32 | 0.209 |
| ≤2000 RMB | 0.44 | 0.18-1.07 | 0.070 | 3.20 | 1.45-7.03 | 0.004 |
| Number of children |  |  |  |  |  |  |
| 0 | 0.79 | 0.33-1.92 | 0.604 | 0.62 | 0.26-1.44 | 0.266 |
| ≥2 | 0.42 | 0.22-0.80 | 0.008 | 0.61 | 0.33-1.14 | 0.123 |
| Nutrition |  |  |  |  |  |  |
| At risk of malnutrition | 4.07 | 2.45-6.76 | <0.001 | 2.08 | 1.26-3.43 | 0.004 |
| Malnourished | 5.97 | 2.24-15.96 | <0.001 | 4.21 | 1.59-11.13 | 0.004 |
| Number of medicine taken |  |  |  |  |  |  |
| 1~4 | 1.19 | 0.64-2.21 | 0.573 | 0.99 | 0.56-1.76 | 0.970 |
| ≥5 | 1.78 | 0.95-3.32 | 0.071 | 0.87 | 0.48-1.58 | 0.651 |
| Pain |  |  |  |  |  |  |
| Yes | 0.91 | 0.56-1.48 | 0.716 | 0.68 | 0.43-1.09 | 0.113 |
| Smoking history |  |  |  |  |  |  |
| Formal | 1.29 | 0.72-2.32 | 0.392 | 2.16 | 1.24-3.79 | 0.007 |
| Current | 1.68 | 0.76-3.72 | 0.204 | 3.15 | 1.49-6.68 | 0.003 |
| Drinking history |  |  |  |  |  |  |
| Current | 1.68 | 0.78-3.64 | 0.188 | 2.13 | 1.01-4.48 | 0.047 |
| Napping |  |  |  |  |  |  |
| Yes | 1.05 | 0.67-1.64 | 0.831 | 0.54 | 0.35-0.83 | 0.005 |

Reference: taking non-frail group as the reference.

Model^a^: unadjusted;

**Supplementary Table 4 Associations of other covariates with frailty status in brushing teeth frequency model^b^ (n=1208)**

|  | **Pre-frail** | | | **Frail** | | |
| --- | --- | --- | --- | --- | --- | --- |
|  | **OR** | **95%CI** | ***p*-values** | **OR** | **95%CI** | ***p*-values** |
| Nutrition |  |  |  |  |  |  |
| At risk of malnutrition | 4.65 | 2.87-7.51 | <0.001 | 1.92 | 1.20-3.06 | 0.006 |
| Malnourished | 6.81 | 2.88-7.50 | <0.001 | 4.32 | 1.67-11.17 | 0.003 |
| Number of medicine taken |  |  |  |  |  |  |
| 1~4 | 1.26 | 2.61-17.82 | 0.420 | 0.96 | 0.56-1.62 | 0.866 |
| ≥5 | 1.68 | 0.72-2.23 | 0.062 | 0.92 | 0.55-1.54 | 0.747 |
| Pain |  |  |  |  |  |  |
| Yes | 1.07 | 0.97-2.89 | 0.766 | 0.76 | 0.49-1.17 | 0.210 |
| Smoking history |  |  |  |  |  |  |
| Formal | 1.12 | 0.68-1.68 | 0.681 | 2.19 | 1.32-3.63 | 0.002 |
| Current | 1.38 | 0.66-2.88 | 0.393 | 2.48 | 1.24-4.96 | 0.010 |
| Drinking history |  |  |  |  |  |  |
| Current | 1.75 | 0.83-3.67 | 0.142 | 2.65 | 1.29-5.44 | 0.008 |
| Napping |  |  |  |  |  |  |
| Yes | 1.07 | 0.70-1.62 | 0.760 | 0.58 | 0.38-0.86 | 0.008 |

Reference: taking non-frail group as the reference.

Model^b^: adjusted for age, sex, residence, education, marital status, economic source, income, and number of children;
